# Advanced Imaging of Intracranial Atherosclerosis: Lessons from Interventional Cardiology

**DOI:** 10.3389/fneur.2017.00387

**Published:** 2017-08-14

**Authors:** Davor Pavlin-Premrl, Rahul Sharma, Bruce C. V. Campbell, J. Mocco, Nicholas L. Opie, Thomas J. Oxley

**Affiliations:** ^1^Department of Medicine and Neurology, Melbourne Brain Centre at the Royal Melbourne Hospital, The University of Melbourne, Parkville, VIC, Australia; ^2^Heart Institute, Cedars Sinai Medical Center, Los Angeles, CA, United States; ^3^Cerebrovascular Center, Mount Sinai Hospital, New York, NY, United States; ^4^Vascular Bionics Laboratory, Melbourne Brain Centre, Department of Medicine, Royal Melbourne Hospital, The University of Melbourne, Parkville, VIC, Australia

**Keywords:** stroke, intracranial atherosclerosis, intracranial stenting, fractional flow reserve, intravascular ultrasound, optical coherence tomography

## Abstract

Intracranial atherosclerosis is a major cause of ischemic stroke. Patients with a high degree of stenosis have a significant rate of stroke despite medical therapy. Two randomized trials of stenting have failed to show benefit. Improving periprocedural complication rates and patient selection may improve stenting outcomes. Fractional flow reserve (FFR), intravascular ultrasound (IVUS), and optical coherence tomography (OCT) are intravascular imaging techniques employed to improve patient selection and stent placement in interventional cardiology. FFR has been shown to improve cardiovascular outcomes when used in patient selection for intervention. Studies of FFR in intracranial atherosclerosis show that the measure may predict which plaques lead to stroke. IVUS is used in cardiology to quantify stenosis and assist with stent placement. Comparisons with histology show that it can reliably characterize plaques. Several case reports of IVUS in intracranial arteries show the technique to be feasible and indicate it may improve stent placement. Plaque characteristics on IVUS may help identify vulnerable plaques. In interventional cardiology, OCT provides excellent visualization of vessel geometry and is useful periprocedurally. Images reliably identify thin-capped fibroatheromas and other plaque features. Case reports indicate that OCT is safe for use in intracranial arteries. OCT can be used to identify perforator vessels and so may be useful in avoiding perforator strokes, a common complication of stenting. Plaque characteristics on OCT may be useful in patient selection.

## Background

Intracranial atherosclerosis is a major cause of ischemic stroke (1). The success of medical therapy in the treatment of severe intracranial atherosclerosis is limited (2–4). It was envisioned that percutaneous stenting of severe intracranial disease may provide a definitive treatment. However, the first two randomized controlled trials of intracranial intervention demonstrated no benefit over medical therapy and were stopped early due to higher than expected stroke rates in the stenting arms (3, 4). It has been argued that stenting outcomes could be improved by improving patient selection and stent delivery (5).

Improving patient selection for percutaneous coronary stenting is the subject of ongoing interest in interventional cardiology. To determine which patients would benefit most from intervention, numerous advanced imaging techniques aiming to identify high-risk, unstable atherosclerotic lesions have been developed (6–8). Some of these techniques have also proved useful in reducing periprocedural complications (9).

This article reviews the current and potential application of advanced cardiac imaging techniques in interventional neuroradiology.

## Intracranial Atherosclerosis and Stroke

Intracranial atherosclerosis is an important cause of ischemic stroke. Gorelick et al. report that it has recently emerged as the most common subtype worldwide (1). This is largely because of its prevalence amongst Asian populations. For example, in China, intracranial atherosclerosis causes 33–50% of stroke and >50% of transient ischemic attacks.

Medical therapy has had limited success in preventing stroke in patients with severe intracranial atherosclerosis. The Warfarin and Aspirin for Symptomatic Intracranial Arterial Stenosis trial compared the use of aspirin with warfarin in patients with high-grade stenosis (50–99%) of a major intracranial artery (internal carotid, middle cerebral, vertebral, or basilar artery) with a primary end-point of ischemic stroke, brain hemorrhage or non-stroke vascular death (2). The primary end-point occurred in 22.1% of patients in the aspirin group and 21.8% in the warfarin group. 15% of patients on aspirin suffered an ischemic stroke in the territory of the stenotic artery, compared with 12% of patients on warfarin.

Percutaneous intracranial stenting has also not been shown to be efficacious. The Stenting and Aggressive Medical Management for Preventing Recurrent Stroke in Intracranial Stenosis (SAMMPRIS) trial, which tested the Wingspan self-expanding stent in patients with recent transient ischemic attack or stroke, attributed to an intracranial stenosis of 70–99%. All patients in SAMMPRIS received aspirin with the addition of clopidogrel for 90 days, a high dose statin, antihypertensives targeting systolic blood pressure <140 mmHg and were enrolled in a lifestyle modification program that managed primary and secondary risk factors (10). The 30-day and 1-year stroke rates in the medical treatment arm were 5.8 and 12.2%, respectively (3). The trial was stopped early due to safety concerns, with a 30-day stroke rate of 14.7% in the stenting group (*n* = 224) compared with 5.8% in the medical management group (*n* = 227). Of the 21 patients who suffered an ischemic stroke within 30 days of stenting, 15 were in perforator territories. Other common complications in the stenting group were subarachnoid hemorrhage (*n* = 6) and delayed intraparenchymal hemorrhage (*n* = 7). The stent success rate (defined as meaning the lesion was accessed and stent placement resulted in <50% residual stenosis) was 92%.

Percutaneous intracranial stenting was also trialed in The Vitesse Intracranial Stent Study for Ischemic Stroke Therapy (VISSIT), a randomized, parallel group trial that compared balloon expandable stent placement with medical therapy (4). It was hoped that the balloon expandable stent (as compared with the over-the-wire exchange stent design used in SAMMPRIS) used in the VISSIT trial would be associated with a lower complication rate. However, an early unplanned analysis was performed once the results of SAMMPRIS became known and found that stenting compared with medical therapy resulted in an increased 12-month risk of added stroke or TIA in the same territory (36.2 vs. 15.1%), and increased 30-day risk of any stroke or TIA (24.1 vs. 9.4%). Patient selection in the VISSIT trial was also based on the degree of stenosis. 20.7% of patients had an unsuccessful procedure due to the stent not being placed because of vessel tortuosity, misplacement across the target stenotic lesion, and/or arterial injury during placement of the supportive system. 46% of patients had a residual stenosis of greater than 20% post-stenting.

The patient selection process undertaken by the SAMMPRIS and VISSIT trials could potentially be improved. Patients’ stroke risk was based on the degree of stenosis on angiography, but other factors thought to influence stroke risk, such as hemodynamic significance of the lesion (11) and plaque morphology (5) were not considered. Factors that may increase the risk of complications such as the presence of perforators were also not part of the selection criteria. There has been considerable debate about how patient selection might be improved by incorporating such factors (5).

The high periprocedural complication rates may also be lowered by improving technical aspects of the stenting procedure. In particular, the VISSIT trial demonstrated a high rate of incompletely successful procedures that may have contributed to poor patient outcomes.

## Lessons from Interventional Cardiology

As was the case in the SAMMPRIS and VISSIT trials, angiography is commonly used in cardiology to assess coronary arteries prior to stent implantation (12). However, angiography is subject to three key limitations that may result in suboptimal selection of patients for stenting. First, angiography alone is unable to determine the physiological significance of stenosis (12). Second, angiography may underestimate the true extent of atherosclerosis and is unable to identify signs of positive remodeling—compensatory enlargement of the vessel wall in response to increased plaque size (13). Finally, luminal angiography is unable to identify plaque characteristics that may predict rupture (7).

Due to these deficiencies of angiography, multiple advanced imaging and physiological assessment techniques have been developed. These include fractional flow reserve (FFR), intravascular ultrasound (IVUS), and optical coherence tomography (OCT). These technologies may be applicable to evaluation and management of intracranial atherosclerosis.

## Fractional Flow Reserve

Fractional flow reserve is a means of evaluating the physiological significance of a coronary artery stenosis. It is defined as the ratio between the distal and proximal pressure of the stenosis at maximum hyperemia (12). Effectively, FFR is the ratio between the maximal achievable coronary flow in the stenotic coronary artery and the maximal flow in the same vessel if it were normal. It is measured by passing a wire equipped with a pressure sensor across the stenosis and then measuring the proximal and distal pressures at rest and at maximal hyperemia. Normal FFR is defined as 0.94 to 1.0, with an FFR <0.75 correlated with ischemia.

The use of FFR in patient selection for coronary intervention has been shown to improve event-free survival, reduce unnecessary percutaneous coronary intervention, and improve cost effectiveness. The FFR vs. Angiography for Multivessel Evaluation (FAME) study trialed deferring intervention in patients with FFR >0.80 and found reduced rates of cardiac events at both 1 and 2 years (6). The FAME II study, which randomized patients with FFR <0.8 to intervention in addition to best medical therapy or to best medical therapy alone, was stopped prematurely due to a difference in death, myocardial infarction, or urgent percutaneous intervention in favor of intervention (14).

Non-interventional imaging suggests that measuring FFR may help predict stroke in intracranial atherosclerosis. A study that used time-of-flight ratios to measure FFR found that the hazard ratio for stroke in the territory of the symptomatic artery with signal intensity ratio <0.9 was 5.2 as compared to signal intensity ratio ≥0.9 (15). Furthermore, Liebeskind and Feldmann performed retrospective computational fluid dynamics analyses on the SAMMPRIS patients and found that only 76/188 (40%) of those in the severe stenosis category had an abnormal FFR (11), indicating that the measure could be explored for patient selection in future.

The feasibility of using interventional techniques to measure FFR in the intracranial arteries has been demonstrated. 20 patients with intracranial atherosclerosis had the stenosis crossed with a pressure guidewire and FFR measured, with no complications attributed to the procedure (16).

## Intravascular Ultrasound

Intravascular ultrasound enables the quantification of stenosis and characterization of plaques and also assists with stent delivery.

Gray-scale IVUS accurately determines vessel dimensions and wall characteristics and can more sensitively identify early atherosclerosis than angiography (13). In interventional cardiology, IVUS is currently employed in cases where it is not clear on angiography alone whether a lesion requires stenting.

Post-processing modalities beyond gray-scale IVUS has been developed and shown to reliably demonstrate qualitative plaque characteristics. IVUS virtual histology enables classification of plaque components into four categories: fibrous tissue, fibrofatty tissue, necrotic core, and dense calcium, a process validated by comparison with histology (7).

Certain lesion characteristics evaluated using IVUS have been shown to be associated with acute coronary syndromes. The Prospective Natural-History Study of Coronary Atherosclerosis (PROSPECT) trial found that several IVUS findings were independent predictors of subsequent major adverse cardiovascular events: plaque burden (defined as plaque and media cross-sectional area divided by external-elastic-membrane cross-sectional area) of 70% or greater, a minimal luminal area of 4.0 mm^2^ or less and the presence of thin-cap fibroatheroma (19).

Beyond assessing plaque vulnerability, IVUS can be used to improve stent placement by identifying stent malapposition and underexpansion. A meta-analysis has shown that IVUS-guided stent placement improves clinical outcomes compared with angiography guided stent implantation, with significant reductions in major adverse cardiovascular events [odds ratio 0.77 (CI) 0.71–0.83, *P* < 0.001] and stent thrombosis (OR 0.59, 95% CI: 0.47–0.73, *P* < 0.001) (20).

Although limited to case reports, current experience of IVUS in the intracranial arteries indicates that it is safe and may be useful in guiding stent placement. In 2006, Wehman et al. published the first report of IVUS use in the intracranial arteries, detailing the cases of two patients (21). In the first case, the patient underwent intracranial stent insertion to treat an occlusive dissection of the left internal carotid artery. IVUS was reportedly useful as the color-flow feature allowed the identification of residual pseudo-lumen flow, and also assisted in making sure there was optimal stent coverage of the lumen. In the second case, the patient underwent stent placement for high-grade restenosis of the basilar artery. IVUS was used to identify the composition and morphological features of restenosis and helped determine that the lesion was safe for stent placement. Takayama et al. used IVUS virtual histology to characterize the plaque and guide stenting in the case of a symptomatic intracranial right vertebral artery stenosis (22). Meyers et al. used IVUS to image the petrous portion of the internal carotid artery and identified an 85% stenosis due to an atherosclerotic plaque with intraplaque hemorrhage, following which stent-supported angioplasty was performed (23).

In addition to the technical benefits demonstrated in the cases above, IVUS may be useful in identifying vulnerable plaques. There has been limited histological investigation of intracranial atherosclerosis, but current evidence suggests that lipid content of plaques is an independent risk factor for stroke (24). Experience in cardiology suggests that IVUS is an effective means of identifying lipid-rich plaques. Majidi et al. showed that there is good correlation between IVUS virtual histology and histopathology in intracranial atherosclerosis by examining vessels postmortem (17). The possibility that lipid-rich plaques identified with IVUS may be more suitable for stenting warrants further exploration.

## Optical Coherence Tomography

Optical coherence tomography is a catheter-based imaging system that is the light analog of IVUS (8). Its chief advantage is increased resolution compared with IVUS, with axial resolution ranging from 12 to 18 µm, compared with 150 to 200 µm for IVUS, and lateral resolution from 20 to 90 µm, compared with 150 to 300 µm for IVUS. The chief drawback is limited tissue penetration, with OCT reaching 1–3 mm and IVUS reaching 4–8 mm. It uses the backscattering of near-infrared light to produce high-resolution *in vivo* images of arteries. The time it takes for emitted light to travel between the target tissue and back to the lens produces a measurable “echo time delay.” Intravascular OCT requires a single fiber-optic wire that emits light and records the reflection while being pulled back and rotating along the artery.

The increased resolution of OCT allows for improved identification of plaque features, particularly high-risk thin-capped fibroatheroma. OCT can be used to reliably directly measure fibrous cap thickness. Kubo et al. imaged the coronary arteries of 30 acute myocardial infarction patients with OCT, IVUS, and coronary angioscopy and found that OCT was superior in identifying plaque rupture, fibrous cap erosion, intracoronary thrombus, and thin cap fibroatheroma (25). Identification of thin fibrous plaques with OCT has been shown to predict plaque progression in coronary arteries (26).

Optical coherence tomography can also demonstrate other potentially important plaque features. Yabushita et al. reported good correlation between histology and OCT in identifying fibrous, fibrocalcific, and lipid-rich plaques (27). OCT can be used to assess collagen content and smooth muscle cell density, features associated with stable plaques (28), as well as macrophage accumulations and neoangiogenesis (29).

Optical coherence tomography is useful in peri-interventional assessment. A case-control study of 335 patients reported improved outcomes with an OCT-guided approach to coronary stent implantation (9). The OCT group had a significantly lower 1-year risk of cardiac death, myocardial infarction, or repeat vascularization (9.6 vs. 14.8%). The authors report that OCT identified multiple serious procedural issues: edge dissection, reference lumen narrowing, stent malapposition, stent underexpansion, and thrombus. These findings led to additional post-OCT interventions being performed in 34.7% of patients who received OCT. In comparison with IVUS, OCT offers improved stent visualization and identification of stent malapposition (30).

It is likely that OCT will be safe to use in the intracranial arteries, as the OCT probe is smaller than the IVUS probe. It can be used to negotiate complex lesions in calcific and tortuous coronary vessels (31).

The safety of OCT use in the intracranial arteries is supported by multiple case reports. Mathews et al. successfully deployed an OCT probe in three patients undergoing diagnostic angiograms, with no complications reported (32). Given et al. described the use of OCT in a patient with vertebra-basilar insufficiency, who developed posterior circulation symptoms 15 h post-stent placement (33). Angiography showed in-stent thrombus and OCT was employed to identify dissection or other possible antecedent causes that may have led to the clot. Images showed a residual stenosis with no other associated abnormal features, obviating the need for further intervention.

These promising initial experiences with OCT in the intracranial arteries indicate that the technology could reduce stenting periprocedural complications as it has with coronary stenting. A particular advantage of OCT in the intracranial arteries is that arteries are surrounded by cerebrospinal fluid rather than myocardium, giving them increased definition. Another unique feature of intracranial vessels seen on OCT are perforator vessels (33). This is relevant to stent placement, as the most frequent stroke identified in the SAMMPRIS trial were perforator strokes thought to be due to perforator occlusion by displaced or disrupted atheromatous debris (34). Visualizing perforators could reduce the risk of perforator infarction.

It is possible that OCT could improve patient selection by identifying vulnerable plaques. OCT imaging of coronary plaques can identify the presence of macrophages and neovascularization, features associated with stroke in intracranial atherosclerosis. The identification of these features, and others as our understanding of intracranial atherosclerosis deepens, may help to identify better stenting candidates.

## Conclusion

Advanced imaging techniques are currently used in interventional cardiology to provide critical anatomical and physiological information about atherosclerotic plaques, as well as to improve stent delivery.

Fractional flow reserve, in particular, has a strong evidence base for improvements in morbidity by refining patient selection for coronary treatment. Similar approaches in intracranial arterial disease appear promising. This hypothesis needs to be tested in further clinical trials.

Initial studies suggest that the intravascular imaging technologies IVUS and OCT are likely safe for use in the intracranial arteries, although further verification is needed. These technologies improve delivery of coronary stents, and so could potentially reduce the significant periprocedural complication rate seen with intracranial stents. Intravascular imaging also shows promise in identifying vulnerable coronary plaques and may be able to improve patient selection by identifying intracranial plaque risk factors associated with stroke.

## Author Contributions

Each author contributed to the paper according to the ICMJE guidelines for authorship in the main manuscript.

## Conflict of Interest Statement

The authors declare that the research was conducted in the absence of any commercial or financial relationships that could be construed as a potential conflict of interest.
